# Pharmacokinetics and Pharmacodynamics of Intramuscular and Oral Betamethasone and Dexamethasone in Reproductive Age Women in India

**DOI:** 10.1111/cts.12724

**Published:** 2019-12-13

**Authors:** Alan H. Jobe, Mark A. Milad, Thomas Peppard, William J. Jusko

**Affiliations:** ^1^ Division of Pulmonary Biology Cincinnati Children's Hospital Medical Center University of Cincinnati Cincinnati Ohio USA; ^2^ Milad Pharmaceutical Consulting LLC Plymouth Michigan USA; ^3^ Certara, Inc. Princeton New Jersey USA; ^4^ State University of New York School of Pharmacy and Pharmaceutical Sciences University of Buffalo Buffalo New York USA

## Abstract

High‐dose betamethasone and dexamethasone are standard of care treatments for women at risk of preterm delivery to improve neonatal respiratory and mortality outcomes. The dose in current use has never been evaluated to minimize exposures while assuring efficacy. We report the pharmacokinetics and pharmacodynamics (PDs) of oral and intramuscular treatments with single 6 mg doses of dexamethasone phosphate, betamethasone phosphate, or a 1:1 mixture of betamethasone phosphate and betamethasone acetate in reproductive age South Asian women. Intramuscular or oral betamethasone has a terminal half‐life of 11 hours, about twice as long as the 5.5 hours for oral and intramuscular dexamethasone. The 1:1 mixture of betamethasone phosphate and betamethasone acetate shows an immediate release of betamethasone followed by a slow release where plasma betamethasone can be measured out to 14 days after the single dose administration, likely from a depo formed at the injection site by the acetate. PD responses were: increased glucose, suppressed cortisol, increased neutrophils, and suppressed basophils, CD3CD4 and CD3CD8 lymphocytes. PD responses were comparable for betamethasone and dexamethasone, but with longer times to return to baseline for betamethasone. The 1:1 mixture of betamethasone phosphate and betamethasone acetate caused much longer adrenal suppression because of the slow release. These results will guide the development of better treatment strategies to minimize fetal and maternal drug exposures for women at risk of preterm delivery.

**Study Highlights**

**WHAT IS THE CURRENT KNOWLEDGE ON THE TOPIC?**

☑ Betamethasone and dexamethasone are the standard of care for women at risk of preterm delivery to decrease respiratory distress syndrome and mortality. Drug choice and dosing have not been optimized to minimize maternal and fetal exposures, although both steroids are used at the same high total dose of 24 mg.

**WHAT QUESTIONS DID THIS STUDY ADDRESS?**

☑ What are the pharmacokinetic (PK) and pharmacodynamic (PD) characteristics of oral and maternal i.m. dexamethasone and betamethasone needed for developing new treatment strategies to minimize drug exposure?

**WHAT DOES THIS STUDY ADD TO OUR KNOWLEDGE?**

☑ The PKs provide high resolution measurements of the drugs in a relevant population of reproductive age South Asian women. The PDs extend the information about corticosteroid effects and highlight the slow release rate and prolonged cortisol suppression from betamethasone phosphate plus betamethasone acetate, the current standard of care in the United States.

**HOW THIS MIGHT CHANGE CLINICAL PHARMACOLOGY OR TRANSLATIONAL SCIENCE?**

☑ These results support dose finding strategies to revise the standard antenatal corticosteroid regimens to decrease maternal and fetal drug exposures.

Betamethasone (Beta) and dexamethasone (Dex) are the fluorinated corticosteroid congeners that have been used since 1972 as antenatal corticosteroid (ACS) treatments for women at imminent risk of preterm delivery before 34 weeks gestational age to decrease neonatal respiratory distress syndrome and mortality by inducing fetal lung and other organ maturation.^1^ A Cochrane review of ACS compared with placebo or no treatment reported a 31% relative reduction in the risk of neonatal mortality.^2^ The World Health Organization (WHO) recommends maternal i.m. treatment with Dex phosphate (DexP) as four doses of 6 mg given at 12‐hour intervals, or the 1 to 1 mixture of Beta phosphate (BetaP) and Beta acetate (BetaA) as two doses of 12 mg given at a 24‐hour interval.^3^ Although not US Food and Drug Administration (FDA) approved for the ACS indication, BetaP plus BetaA is used preferentially in the United States; DexP is predominantly used in low resource countries and is widely available; and BetaP as two doses of 12 mg given at a 24‐hour interval is used in the United Kingdom.^4^ BetaP plus BetA is not available in the United Kingdom, and BetaP is not available for i.m. use in the United States. Drug coverage is as high as 90% of women at risk of preterm delivery in high resource environments.^5^ However, due in part to unproven efficacy and poor drug availability, drug coverage is very low in many low resource environments with limited maternal and newborn care.^6^

Dose‐ranging trials were never performed to evaluate efficacy or safety because the indication is off label and the drugs are readily available and inexpensive.^4^, ^5^ However, these potent corticosteroids have significant complications associated with routine short‐term use.^7^ Dosing for ACS is indirect because the pregnant woman is given the phosphorylated or acetylated prodrugs to achieve a benefit for the premature newborn with the time from treatment to preterm delivery often predicted poorly. Our group and others have demonstrated in animal models that the high‐dose treatments in common use may expose the fetus to excessive amounts of the steroids.^8^, ^9^ BetaP or DexP given i.m. or orally to the mother are rapidly dephosphorylated to yield high maternal and fetal concentrations that may not be necessary for the fetal lung maturation and, thus, may only contribute to fetal toxicity.^10^, ^11^, ^12^ All recommended ACS treatments are given maternal i.m., although oral preparations of these steroids are widely available and were recently shown to be comparably effective in preterm sheep and primate models.^12^, ^13^

The pharmacokinetics (PKs) of ACS have been assessed in pregnant rats and sheep, but not in humans.^8^, ^14^, ^15^ Furthermore, the available information about the PKs and pharmacodynamics (PDs) of these drugs in humans is dated, incomplete, and based on analytical assays that were not sensitive at the low concentrations that are effective for fetal lung maturation. As more is now known about fetal maturational responses to antenatal steroids from animal models,^9^, ^16^ we report high resolution PK and PD evaluations of Beta and Dex in healthy reproductive age women in India to support the development of novel ACS regimens for use in low and middle‐income countries and potentially for worldwide use. We evaluated single clinical doses of DexP and BetaP given i.m. and orally in comparison to the clinical treatment with intramuscular BetaP plus BetaA.

## Methods

### Study design

We performed an open label, randomized, two‐period crossover study in healthy reproductive age women who were fasted overnight before each corticosteroid treatment. The randomization included eight treatment sequences of 6 subjects each for a sample size of 48 subjects to receive 96 treatments for measuring PK and PD (**Table** 1). The subjects were admitted to the Syngene International research facility in Bangalore, India, for the period 1 treatment for dinner and an overnight fast prior to a 24‐hour baseline sampling period beginning at 8am. The participants then had normal meals, another overnight fast, and the drug treatment at 8am followed by timed interval blood draws to 96 hours. The subjects were discharged from the research facility for 14 days after the period 1 treatment. They were readmitted for dinner and a fast prior to a second treatment and 96 hours of blood draws. The protocol was approved by the ACE Independent Ethics Committee, Bangalore India, on May 30, 2018, and by the Institutional Review Board at Cincinnati Children's Hospital Medical Center (CCHMC 2018‐3878) on July 25, 2018. The protocol was listed on http://ClinicalTrials.gov (NCTO3668860, September 18, 2018). Subjects were randomized in four cohorts between September 20 and October 30, 2018.

**Table 1:** Assignments for treatments to delivery with 6 mg dexamethasone or betamethasone maternal i.m. or 0.5 mg oral tablets separated by a 14 day washout period from the initial treatment in period 1

Period 1	Period 2	Number randomized	Number completed
Dexamethasone NaPO4 i.m.	Betamethasone NaPO4 i.m.	6	6
Betamethasone NaPO4 i.m.	Dexamethasone NaPO4 i.m.	6	6
Betamethasone NaPO4 plus betamethasone acetate i.m.	Dexamethasone NaPO4 oral	6	5
Dexamethasone NaPO4 oral	Betamethasone NaPO4 plus betamethasone acetate i.m.	6	6
Betamethasone NaPO4 oral	Dexamethasone NaPO4 oral	6	5
Dexamethasone NaPO4 oral	Betamethasone NaPO4 oral	6	5
Betamethasone NaPO4 plus betamethasone acetate i.m.	Betamethasone NaPO4 oral	6	6
Betamethasone NaPO4 oral	Betamethasone NaPO4 plus betamethasone acetate i.m.	6	6

### Subjects

Volunteers were screened with physical examinations, routine blood tests, chest x‐rays, electrocardiograms, and health questionnaires to identify healthy, literate, reproductive age women. Subjects who were nonsmokers or moderate smokers, nondrinkers or occasional drinkers who abstained for the period of the study, and were not pregnant and using contraception qualified for inclusion in the study. Women with a history or prescreening that identified substantial disease, history of drug abuse, recent medication use, such as monoamine oxidase inhibitors or caffeine, were excluded from the study. The women were individually consented in English or their native language.

### Drug treatments

We used doses of the sodium salts of the phosphorylated drugs to deliver 6 mg Beta, 6 mg Dex, or 6 mg of a 1:1 mixture of BetaP plus BetaA to deliver 3 mg Beta from each prodrug. The corticosteroid drugs, sources, and integrities are given in **Table** **S1**. Beta or Dex contents of the formulations were measured by mass spectrometry by Exemplify, Cranbury N.J. The BetaP plus BetaA as Celestone Soluspan (Merck, Kenilworth, NJ) was permitted into India only for this protocol.

### Sample handling and analyses

For period 1 and following an overnight fast, the 24‐hour baseline blood samples were drawn beginning at 8:00am at 0, 1, 2, 3, 4, 6, 9, 11, 15, and 24 hours. The subjects were again fasted overnight prior to the drug treatment followed by blood draws at 0.5, 1.0, 1.5, 2.0, 3.0, 4.0, 6.0, 12, 18, 24, 30, 36, 48, 60, 72, and 96 hours post‐treatment. After the washout period, the same sampling schedule was followed as in period 1. All blood was drawn into K2EDTA anticoagulant tubes, with addition of 0.1 mM Na_2_HAsO_4_ to the tubes used for PK analyses to prevent dephosphorylation of the prodrugs in plasma at early times after treatment.^17^ Chilled blood samples were centrifuged within 30 minutes at 4°C to recover plasma, which was frozen and stored at −70°C. Syngene developed a liquid‐chromatography tandem mass spectrometry assay with a sensitivity of 0.1 ng/mL for Beta and Dex. Plasma cortisol with deuterated internal standards was also measured by liquid‐chromatography tandem mass spectrometry analyses with a sensitivity to 1 ng/mL. Plasma glucose was measured by the glucose oxidase method. Blood neutrophils and basophils were counted with an SY5MEX XN 1000 hematology analyzer. T helper and T suppressor cells were measured by automated flow cytometry with a Beckman Coulter Navioz flow cytometer using CYTO‐STAT tetra CHROME CD45‐FITC/CD4‐RD1/CD8‐ECD/CD3‐PC5 and CYTO‐STAT tetra CHROME monoclonal antibody reagents.

### Statistics

PK parameters for Dex and Beta were calculated using Phoenix WinNonlin version 8.1. Rebound Times (RTs) were calculated for each PD for each subject, defined as the time from drug administration to the return of the variable to its baseline value, where the baseline was a series of time‐matched samples collected during the 24 hours prior to dosing in period 1 to account for circadian rhythms in the PD variables. The baseline curve from period 1 for each subject was used as the baseline for period 2. The change from baseline in the area under the exposure curve from dosing to rebound time (ΔAUEC_RT_) was calculated by subtracting the baseline AUEC_RT_ (defined as the area for the baseline period, extrapolated to RT) from the AUEC_RT_. Summary statistics are presented for PK parameters. Due to the large number of subjects with right censored values for RT and ΔAUEC_RT_, Kaplan–Meier estimates of the median, 25th, and 75th percentiles were determined. When there was too much censoring to determine even the Kaplan–Meier estimate of a percentile, a lower boundary was estimated by imputing the individual RT or ΔAUEC_RT_ using the latest observed value in the same period. These lower bound estimates are indicated with a greater than sign (>). Inferential statistics are not reported because the objectives of the trial were to characterize the PK and PD profiles of the different regimens rather than to demonstrate either equivalence or differences with a prespecified level of precision.

## Results

The study was a randomized, open–label, two period crossover comparison of the drugs following an overnight fast (**Table** 1). The subjects were a remarkably uniform group of 48 Indian Asian women with similar mean ages, weights, heights, and body mass index (BMI; **Table** **S2**). Three subjects (6%) were withdrawn from the study prematurely. One subject had vomiting in period 2, and a second subject did not return for the second treatment period. A third subject had a serious adverse event of cellulitis and sepsis associated with a catheter used for blood draws during period 1. Other adverse events were minor and did not impact the study.

### PKs of Dex and Beta

**Figure 1:**
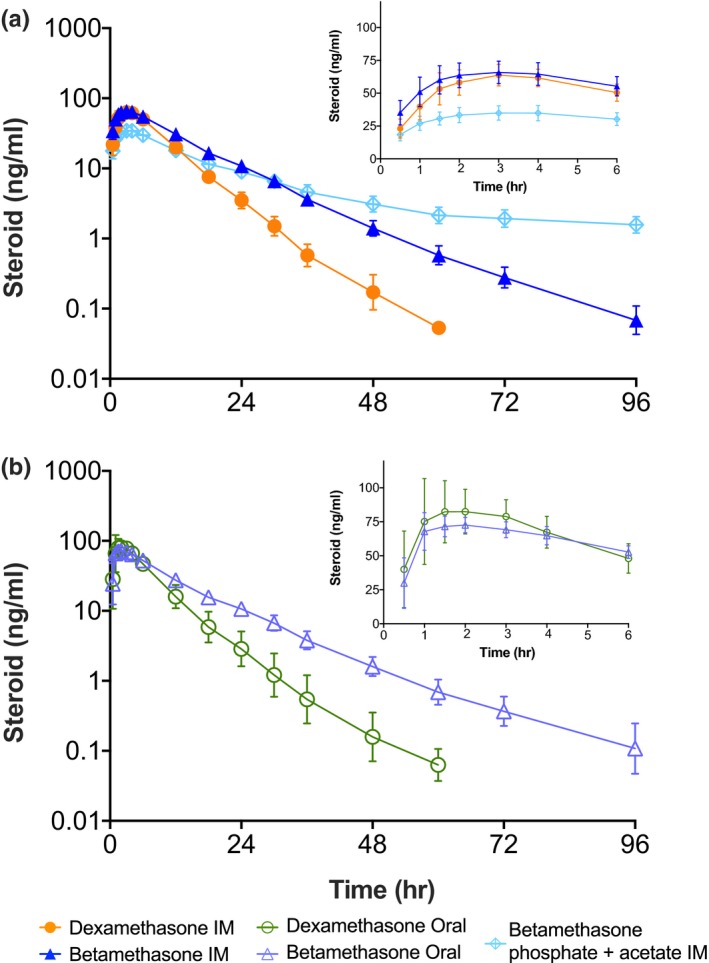
Plasma concentration‐time profiles following 6 mg doses given i.m. of (**a**) dexamethasone phosphate (DexP), betamethasone phosphate (BetaP), and BetaP plus betamethasone acetate (BetaA) as semi‐log plots of geometric means ± 1 SD vs. time. The insert gives the concentration time profiles on a linear scale as arithmetic means ± SD. Frame (**b**) gives concentration‐time profiles following oral administration of 6 mg DexP and BetaP.

The geometric mean ± 1 SD corticosteroid plasma concentrations for the 6 mg Dex i.m. and 6 mg Beta i.m. treatments demonstrate minimal variability for the large number of curves analyzed (**Figure** 1 **a**). The peak plasma concentration (C_max_) and time of maximum plasma concentration (T_max_) values are comparable for the two drugs (**Table** 2). The terminal half‐life (t_1/2_) value for BetaP is twice as long as for DexP, and the differences in the AUC_24_ or AUC_96_ values result from the lower apparent clearance (CL/F) for BetaP. BetaP plus BetaA has a multiphasic concentration‐time profile due to the mixture of the fast release BetaP and slow release BetaA, with a C_max_ of 35.4 ng/mL, about 50% of BetaP or DexP C_max_, and a T_max_ of 3 hours, similar to BetaP. For the 12 participants who received BetaP plus BetaA in period 1, the plasma Beta level is 1.5 ± 0.3 ng/mL at 96 hours. After the 14‐day washout between treatments, Beta is still measurable with a mean value of 0.34 ± 0.22 ng/mL.

**Table 2:** Mean ± 1 SD PK parameters following a single dose of 6 mg corticosteroid

Treatment	I.M. dexamethasone phosphate	I.M. betamethasone phosphate	I.M. betamethasone phosphate plus betamethasone acetate	Oral dexamethasone phosphate	Oral betamethasone phosphate
Number of PK curves	12	12	24	22	23
C_max_ (ng/mL)	65.0 ± 8.0	67.6 ± 8.9	35.4 ± 5.6	95.3 ± 15.9	76.8 ± 7.2
T_max_ (hour)^a^	3.0 (1.5–4.0)	3.0 (1.0–4.0)	3.0 (1.0–4.0)	1.5 (1.0–4.0)	1.5 (1.0–3.0)
AUC_0–24_ (ng hour/mL)	617 ± 88	811 ± 112	473 ± 74	661 ± 142	796 ± 74
AUC_0–96_ (ng·hr/mL)	643 ± 94	942 ± 130	701 ± 118	688 ± 160	938 ± 106
CL/F (mL/hour)	9,471 ± 1,139	6,466 ± 805	7,404 ± 1,598	9,156 ± 2,034	6,443 ± 699
V/F (mL)	70,961 ± 10,131	94,584 ± 23,539	582,583 ± 298,893	70,606 ± 12,389	127,530 ± 70,360
t_1/2_ (hour)	5.2 ± 0.4	10.2 ± 2.5	59 ± 35	5.5 ± 1.2	13.9 ± 7.5

AUC_0–24_, 0–24‐hour area under the concentration‐time curve; AUC_0–96_, 0–96‐hour area under the concentration‐time curve; CL/F, total apparent clearance; C_max_, peak plasma concentration; PK, pharmacokinetic; t_1/2_, terminal half‐life; T_max_, time to peak plasma concentration; VF, volume fraction.

^a^ Values as median and range.

The oral dosing with DexP and BetaP results in C_max_ values that are similar to each other, but both are higher than for i.m. treatments. Oral T_max_ values are shorter (median 1.5 hours) than for the i.m. treatments (**Figure** 1 **b**). The mean AUC_96_ for oral and IM DexP are similar to one another (688 and 643 ng hour/mL), suggesting similar relative bioavailability. Mean AUC_96_ also are similar for oral and i.m. BetaP (938 and 942 ng hour/mL), but lower (701 ng hour/mL) for BetaP plus BetaA. The t_1/2_ following oral or i.m. administration are shorter for Dex (about 5.5 hours) than Beta (about 11 hours).

### PDs

#### Plasma glucose

**Figure 2:**
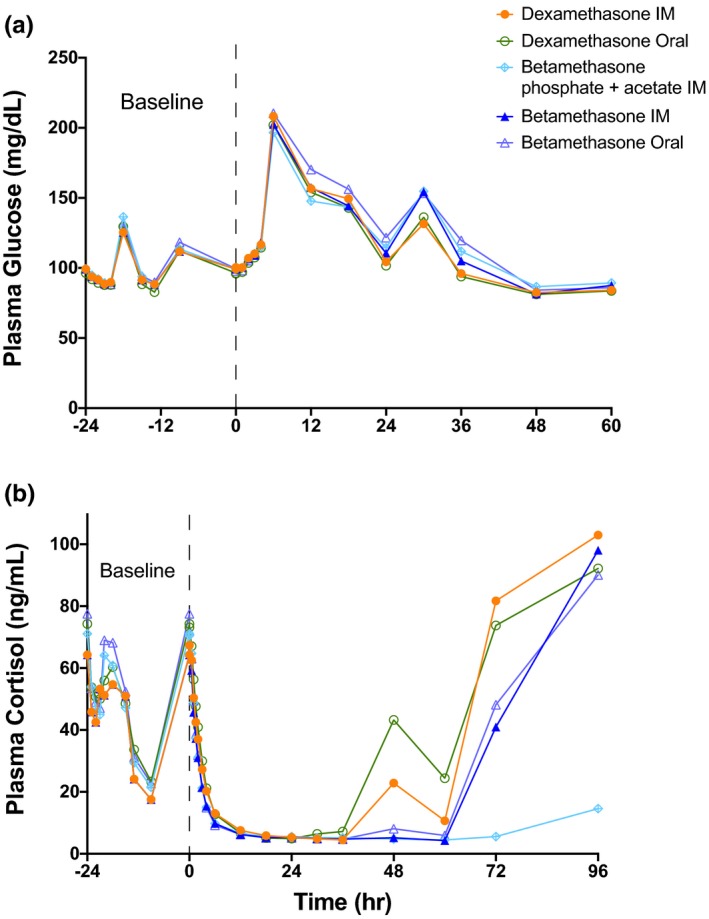
Pharmacodynamics of plasma glucose and cortisol following the steroid treatments. (**a**) Mean values for plasma glucose measured for the 24 hour baseline period and for 60 hours following treatment at hour 0. (**b**) Mean values for cortisol demonstrate the diurnal changes in cortisol for 24 hours prior to the steroid treatment at hour 0. All treatments caused severe adrenal suppression with variable times of recovery for measurements to 96 hours.

Baseline mean plasma glucose values for the 24 hours before steroid treatment were similar across the five treatment groups (**Figure** 2 **a**). **Figure** **S1** **a** gives mean measurements ± 1 SD. Blood glucose increased with the corticosteroid treatments similarly from the fasting baseline mean of about 100 mg/dL to a mean of about 200 mg/dL in association with lunch (**Table** 3). The corticosteroid effect on glucose was larger than the postprandial increases during the baseline period. The median times required for glucose measurements to rebound to baseline values (RT) were 33.1 and 30.0 hours for the two DexP treatments, and ranged from 36.1–37.7 hours for the three Beta treatments. The subsequent 8:00am glucose at 24 hours was significantly elevated for the five groups relative to the time‐matched baseline, as was the glucose associated with lunch on day 2. Thus, a single dose of 6 mg BetaP or DexP had a large effect on plasma glucose for 24 hours with residual increases into day 2. Excess glucose attributed to steroid exposure, expressed as the increase from baseline in the area under the exposure curve from hour 0 to the RT (ΔAUEC_RT_), had median values ranging from 1,191–1,532 mg hour/dL across the five treatments.

**Table 3:** Summary statistics (mean ± SD or median, 25th–75th percentiles) of PD parameters for glucose and cortisol following a single dose of 6 mg corticosteroid

Treatment	I.M. dexamethasone phosphate	I.M. betamethasone phosphate	I.M. betamethasone phosphate plus betamethasone acetate	Oral dexamethasone phosphate	Oral betamethasone phosphate
Number of PD curves	12	12	24	22	23
Glucose					
Hour 0 fasted (mg/dL)	100.4 ± 11.2	99.7 ± 7.7	99.7 ± 12.5	96.0 ± 6.4	97.8 ± 10.5
C_max_ (mg/dL)	209.4 ± 38.5	211.9 ± 42.6	199.9 ± 34.4	203.6 ± 29.3	213.5 ± 31.0
T_max_ (hours)	6.0 6.0–6.0	6.0 6.0–9.0	6.0 6.0–6.0	6.0 6.0–6.0	6.0 6.0–6.0
Number censored	0	1	0	0	0
RT (hours)	33.1 23.9–37.7	36.1 30.0–41.6	36.9 30.0–38.4	30.0 23.8–36.8	37.7 29.5–41.7
Increase AUEC_RT_ (mg hour/dL)	1,243 992–1,511	1,394 1,031–1,618	1,253 942–1,501	1,191 964–1,524	1,532 1,006–2,025
Cortisol					
Hour 0 (µg/mL)	67.4 ± 15.7	65.0 ± 19.8	70.5 ± 33.6	73.2 ± 19.5	72.0 ± 24.3
C_min_ (µg/mL)	4.4 ± 1.1	4.2 ± 1.1	4.4 ± 1.3	4.1 ± 1.3	4.1 ± 1.0
T_min_ (hours)	36.0 36.0–36.0	54.0 36.0–60.0	60.0 60.0–60.0	36.0 30.0–36.0	60.0 36.0–60.0
Number censored	0	0	20	0	0
RT (hours)	61.5 60.6–63.5	72.6 62.8–79.4	> 96 > 96–> 96	62.3 50.7–62.8	73.5 63.0–82.2
Decrease AUC_RT_ (µg hour/mL)	1,761 1,600–1,899	2,143 1,960–2,595	> 3,985 3,985–> 4,039	1,744 1,622–2,210	2,522 2,260–3,393

Subjects whose cortisol or glucose did not rebound by the latest measurement in that period have a censored rebound time and censored AUEC_RT_. Medians, 25th percentiles, and 75th percentiles are based on Kaplan–Meier estimates when censoring is present, and when censoring is too extensive for those to be determined, a lower limit is presented (prefaced with a “>”) based on the latest available measurement in that period.

AUEC_RT_, baseline in the area under the exposure curve from dosing to rebound time; C_max_, peak plasma concentration; C_min_, minimum plasma concentration; PD, pharmacodynamic; RT, rebound time; T_max_, time to peak plasma concentration; T_min_, time to minimum plasma concentration.

#### Plasma cortisol

The average am plasma cortisol was about 70 µg/mL with a normal circadian rhythm (**Figure** 2 **b**, **Table** 3). Means ± SD are given in **Figure** **S1** **b**. The five drug treatments caused rapid and similar decreases in cortisol to a mean nadir of about 4 µg/mL. Median RT and decreases in AUEC_RT_ were similar for oral and i.m. DexP, about 60 hours and 1,750 µg hour/mL. For the oral and i.m. BetaP treatments, the median RT was about 72 hours, and the median decrease in AUEC_RT_ values were 2,143 and 2,522 µg hour/mL, respectively. For BetaP plus BetaA, the RT was > 4 days in 20 of the 24 subjects, and the median reduction in AUEC_RT_ was in excess of 3,985 µg hour/mL. The low but measurable values in 10 subjects of Beta 14 days after treatment in period 1 with BetaP plus BetaA had no significant effect on the subsequent treatments in period 2. Mean changes in cortisol from period 1, hour 0 to period 2, hour 0 are summarized in **Table** **S3**, and were not significantly different among the five treatments (*P* = 0.637).

#### Blood neutrophils

**Figure 3:**
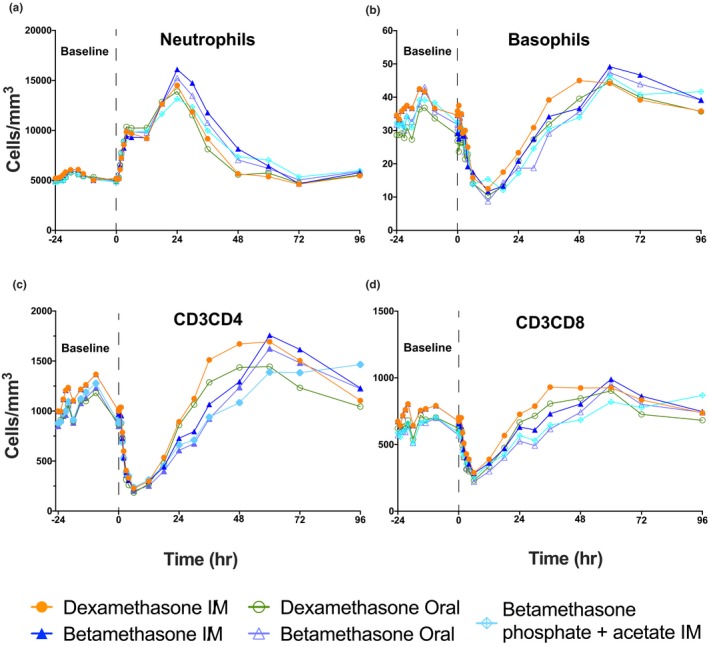
Pharmacodynamics of blood neutrophils, basophils, and CD3CD4 and CD3CD8 lymphocytes. Mean response curves giving the baseline measurements for the 24 hours before the 6 mg corticosteroid treatments given at hour 0. The treatments are color coded, with measurements to 96 hours. (**a**) Neutrophils, (**b**) Basophils, (**c**) CD3CD4 lymphocytes, and (**d**) CD3CD8 lymphocytes.

Mean blood neutrophil counts were remarkably similar for the five treatment groups during the 24‐hour baseline (**Figure** 3 **a**, with individual group mean curves ± SD in **Figure** **S2** **a**). The mean 8:00am (hour 0) neutrophil count on the day of dosing was ~ 5,000/mm^3^ and increased to about 15,000/mm^3^ for all treatments after about 24 hours (**Table** **S4**). The median RTs were shorter for i.m. DexP at 49.4 hours than for oral DexP at 46.1 hours than for the oral and IM BetaP and BetaP plus BetaA treatments, respectively (63.5–74.6 hours). Similarly, median increases in AUEC_RT_ were lower for i.m. and oral DexP (228 and 203 × 10^3^ cells hour/mm^3^, respectively) than for the BetaP and BetaP plus BetaA treatments (267 to 303 × 10^3^ cells hour/mm^3^). There was no evidence of a carryover effect from BetaP plus BetaA from period 1 on the period 2 corticosteroid mediated increase in neutrophils (**Table** **S5**).

#### Blood basophils

The five corticosteroid treatments decreased basophil counts similarly over time (**Figure** 3 **b**; individual group curves in **Figure** **S2** **b**), with baseline mean values ranging from 26.8–35.8 cells/mm^3^, and decreasing to a nadir of 7.4–9.8 cells/mm^3^ between hours 6 and 12 post‐treatment. Median RT and reductions in AUEC_RT_ were similar among the treatment groups, ranging from 26.3–30.0 hours and 471–553 cells hour/mm^3^, except for the oral DexP treatment, which had a smaller reduction in AUEC_RT_ of 259 cells hour/mm^3^ (**Table** **S4**).

#### Blood CD_3_CD_4_ lymphocytes (helper T cells)

Blood CD_3_CD_4_ lymphocytes were decreased similarly by the five corticosteroid treatments from mean baseline values of 864–1,011 cells/mm^3^ to mean nadirs of 175–235 cells/mm^3^ after 6 hours of treatment (**Figure** 3 **c** **,** **Table** **S4** **, and** **Figure** **S2** **b**). The RTs were shorter and AUEC_RT_ lower for the DexP than for the BetaP or BetaP plus BetaA treatments. Median RTs were ~ 25 hours for Dex P, and 40–42 hours for BetaP or BetaP plus BetaA. Median reductions in AUEC_RT_ were 17.4 and 15.5 × 10^3^ hour/mm^3^ for i.m. and oral Dex P, respectively, and ranged from 19.4–21.8 × 10^3^ hour/mm^3^ for the BetaP or BetaP plus BetaA treatments.

#### Blood CD_3_CD_8_ lymphocytes (suppressor T cells)

The five corticosteroid treatments rapidly decreased blood CD_3_CD_8_ cell counts from an average of 615–702 cells/mm^3^ to a nadir of 213–287 cells/mm^3^ by 6 hours (**Figure** 3 **d** **,** **Table** **S4** **, and** **Figure** **S2** **d**). Median RT was longer for BetaP plus BetaA (28.6 hours) and oral BetaP (29.8 hours) than for the other three treatments (22.6–24.0 hours). The i.m. Beta treatment had the greatest decrease in AUEC_RT_.

### Rebound times and ΔAUEC comparisons

**Figure 4:**
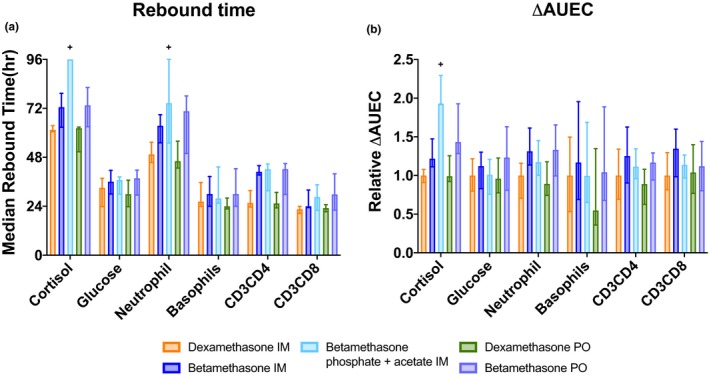
Bar graphs of median ± 25th–75th percentiles of rebound times (RTs) and relative potency using the Kaplan–Meier method to account for observations that are right censored due to a failure to rebound from the corticosteroid treatment effect prior to the last observation. Plus signs are used to indicate when there was too much censoring to determine the 75th percentile per the Kaplan–Meier method, and instead a lower bound is presented, imputed with the final observations of the individually censored subjects. (**a**) RTs were calculated as the time (hours) that the corticosteroid treatment effect returned to the time‐matched baseline value, calculated for each individual measurement. (**b**) Relative potency was calculated as the change from baseline in the area under the exposure curve (ΔAUEC) from hour 0 to the RT, normalized to the median value for DexP IM. To enhance readability, both increases and decreases in ΔAUEC are shown as positive numbers. Glucose and neutrophils increased with corticosteroid exposure, whereas cortisol, basophils CD_3_CD_4_, lymphocytes, and CD_3_CD_8_ lymphocytes decreased.

RTs were consistently shorter across the PD measurements for oral and i.m. Dex than Beta, with remarkably longer RT for BetaP plus BetaA for cortisol and neutrophils (**Figure** 4). Thus, the BetaA component had long lasting PD effects even when given at the dose of 3 mg. The pattern of increased ΔAUEC for Beta relative to Dex reflected the longer t_1/2_ for Beta.

## Discussion

This study resulted from our PK and PD measurements in sheep and monkey preterm birth models indicating that current ACS treatments likely are exposing mother and fetus to high blood levels of the steroids for longer than necessary.^11^, ^13^, ^16^ This study in reproductive age nonpregnant Indian Asian women evaluated the PK and PD responses to 6 mg DexP i.m., as it is the most commonly used regimen in low resource environments when given four times, 12 hours apart.^3^ To better understand other treatment options, we also evaluated 6 mg BetaP and BetaP plus BetaA, but at the 6 mg dose rather than the clinical dose of 12 mg. Relative to older reports, we used collection methods that stabilized the phosphate, assays for the steroids with lower sensitivities (0.1 ng/mL), and measured plasma drug levels and PD responses to 96 hours to extend previous observations.^18^ The study had a similar crossover design as used by Mager *et al*. to evaluate variability of corticosteroid responses between subjects.^18^

The major differences between Beta and Dex were the CL/F and longer t_1/2_ for Beta, independent of oral or i.m. route. The CL/F and longer half‐life for Beta is consistent with the different intrinsic clearance assessed in human liver microsomes where the Dex clearance is 1.89‐fold greater than Beta (unpublished). The finding is also similar to that observed in monkeys where Dex total clearance is 1.7‐fold greater than Beta.^12^ The AUC values for i.m. and oral BetaP were comparable as were the AUC values for i.m. and oral DexP. Both BetaP regimens with CL/Fs had an average of 35% greater exposure AUC than DexP. The initial Beta and Dex plasma levels increased to a higher C_max_ more rapidly for oral dosing than for i.m. dosing (median 1.5 vs. 3.0 hours). In contrast, two reports indicate T_max_ at about 2 hours for oral Dex and 1 hour for i.m. Dex in pregnant women.^19^, ^20^ However, Queckenberg *et al*.^21^ reported maximal peak concentrations at 1 hour for oral Dex in fasted adults. Another variable confounding virtually all previous clinical PK studies is that at early times plasma will contain the prodrugs, BetaP or DexP. These prodrugs will be rapidly dephosphorylated after the blood is drawn unless the conversion is blocked with arsenate, as was done in the present study.^17^, ^22^

The concentration‐time profiles following BetaP plus BetaA reflect rapid release of the phosphate component at early times and slow release for 2 weeks or more from the depo BetaA component. Of note, this single dose of 3 mg Beta as BetaA is 25% of the dose normally used for ACS. BetaA as single component drug for i.m. use is not available. The very long duration of steroid exposure may be undesirable for both mother and fetus.

Strengths of this study are the multiple PD measurements to evaluate comparability of the drugs *in vivo* (**Figure** 4). The large PD effects for all variables will be aggravated by the repeated doses for ACS in pregnant women, particularly for the glucose intolerance associated with pregnancy. Pregnant women treated with BetaP plus BetaA and monitored with continuous glucose monitors had glucose elevations for 2 days.^23^ Pregnant women with BMI < 30 had blood glucose levels > 140 mg/dL for 15 hours following the BetaP plus BetaA, with lower glucose values for women with BMI > 30.^24^ In this population of low weight women with an average BMI of 24, the five single treatments increased blood sugar to about 200 mg/dL at 6 hours, with a return to the baseline value after about 30 hours. The glucose curves overlapped for the five corticosteroid treatments indicating equivalent effects on blood sugar despite the differences in PK for DexP and BetaP. This glucose value is higher than for the report for women in labor,^24^ probably because of the lower weight and BMI of the women in this study. Maternal hyperglycemia will cause hypoglycemia in newborns, which increased from 15% to 24% in a trial of ACS for late preterm pregnancies.^25^ In low resource environments, newborn hypoglycemia may not be effectively monitored or treated, and may cause neurodevelopmental injury.^26^, ^27^

Short‐term use of oral corticosteroids is frequent worldwide with underappreciated risks of sepsis, venous thrombosis, and fracture.^7^ Such adverse effects on the mother have not been closely monitored for the generally healthy pregnancy population. Another clinical concern from animal models is the fetal programming that can result from exposure to corticosteroids.^28^ The 6 mg oral or i.m. dexamethasone dose caused severe adrenal suppression for about 60 hours and for significantly longer (72 hours) with oral or i.m. Beta. The repeated doses of ACS used clinically should cause about 5 days of adrenal suppression in stressed women who often have infection associated with preterm labor. A meta‐analysis of clinical trials conducted in mostly high resource settings did not identify an elevated risk of maternal or fetal infections from ACS.^2^ However, the largest trial of an ACS intervention in low‐income and middle‐income countries reported a 67% increase in the odds of maternal infection in clusters randomized to the intervention, where 45% of the women delivering low birthweight infants received four doses of Dex 6 mg, compared with control facilities where only 10% received ACS.^29^ In the present trial, the BetaP plus BetaA regimen caused even longer adrenal suppression. Our results showing sustained concentrations of Beta following BetaP plus BetaA are not unique but are underappreciated by the field.^30^

Although generally not considered as relevant to ACS therapy, i.m. BetaP and DexP are known to cause large effects that are associated with immune suppression.^31^ In a meta‐analysis, maternal neutrophils increased with ACS and stayed elevated for the duration of treatment to a high of 18,300 mm^3^.^32^ The 6 mg dose of Dex or Beta increased neutrophils to comparable peak levels of about 15,000 mm^3^ at 24 hours, and with a significantly longer RT for Beta. The decreases in basophils were similar for the three drugs. Corticosteroids also had profound effects to decrease CD_3_CD_4_ helper T cells and CD_3_CD_8_ suppressor T cell lymphocytes in the blood. There were not differences of note between the two corticosteroids by oral or i.m. route, and lymphocyte suppression was also comparable for the BetaP plus BetaA. A concern for clinical use is that corticosteroids have large effects on the developing immune system of the fetus that could alter immune function in the child and adult.^33^, ^34^, ^35^

The study has limitations as we studied only fasted healthy Indian‐Asian women. Extrapolation to other populations of different racial backgrounds, a wide range of BMI, nonfasted, and pregnant women must be done with caution. Studies to mimic ACS use in practice will also require multiple doses. Comparable intensive studies in pregnant women at risk of preterm labor are impractical, but these results can guide limited sampling of at‐risk pregnancies to test our observations in clinical settings. Another limitation was that subjects given BetaP plus BetaA were not followed beyond 96 hours to measure prolonged cortisol, or neutrophil RTs. This report includes the types of studies that should have been done 40 years ago to optimize ACS regimens prior to becoming standard of care. This basic information together with PK and PD information on different populations need to be considered within the context of practical dosing intervals, drug availability, and treatment route for dosing to minimize fetal exposures.

In summary, we report high resolution PK and PD measurements of clinical doses of ACS in healthy, nonpregnant, reproductive age women with normal BMI. These results are unique because we used more sensitive Beta and Dex assays and continued the measurements for 96 hours to capture late drug exposures and effects. We report observations about the very long persistence of Beta from BetaP plus BetaA in plasma and prolonged adrenal suppression. The adverse immune implications from the PD effects of BetaP or DexP given orally or i.m. have not been discussed by the field. The three drugs have similar PD exposure‐response effects, but their different PK profiles should guide clinical use.

## Funding

This work was funded by a grant to A.J. from the Bill & Melinda Gates Foundation (OPP1189571). Trial support and statistical and analytical support to M.M. and T.P. was by contract from the Bill & Melinda Gates Foundation. W.J. is supported by National Institutes of Health (NIH) grants GM24211 and GM131800.

## Conflict of Interest

The authors declared no competing interests for this work.

## Author Contributions

All authors wrote the manuscript, designed the research, and analyzed the data.

## Supporting information


**Figure S1.** Baseline and treatment response curves for (a) plasma glucose and (b) plasma cortisol. Group means ± 1 SD are given for each of the IM or Oral treatments that delivered 6 mg of dexamethasone or betamethasone.

**Figure S2.** Baseline and treatment response curves for (a) blood neutrophils, (b) basophils, (c) CD3CD4 lymphocytes, and (d) CD3CD8 lymphocytes. Group means ± 1 SD are given for each of the IM or Oral treatments that delivered 6 mg of dexamethasone or betamethasone.

**Table S1.** Corticosteroids and source used for the study.

**Table S2.** Demographics of study population.

**Table S3.** Baseline cortisol values and Percent change from Period 1 to Period 2.

**Table S4.** Pharmacodynamic Values for Neutrophils, Basophils, CD3CD4 and CD3CD8 lymphocytes in blood following 6mg corticosteroids treatments.

**Table S5.** Neutrophil counts and Percent change from Period 1 to Period 2.

**Supplemental Figure Legends.** Supplemental Figure Legends.

**Supplemental cover Page.** Supplemental cover page.
