# Supplementary material for: Pharmacokinetics and Pharmacodynamics of Intramuscular and Oral Betamethasone and Dexamethasone in Reproductive Age Women in India
Source: Clin Transl Sci. 2019 Dec 13;13(2):391–9. doi: 10.1111/cts.12724 (PMC7070803; doi:10.1111/cts.12724)
Supplement: Supplementary file 1 — Figure S1. Baseline and treatment response curves for (a) plasma glucose and (b) plasma cortisol. Group means ± 1 SD are given for each of the IM or Oral treatments that delivered 6 mg of dexamethasone or betamethasone. [file CTS-13-391-s001.pdf]

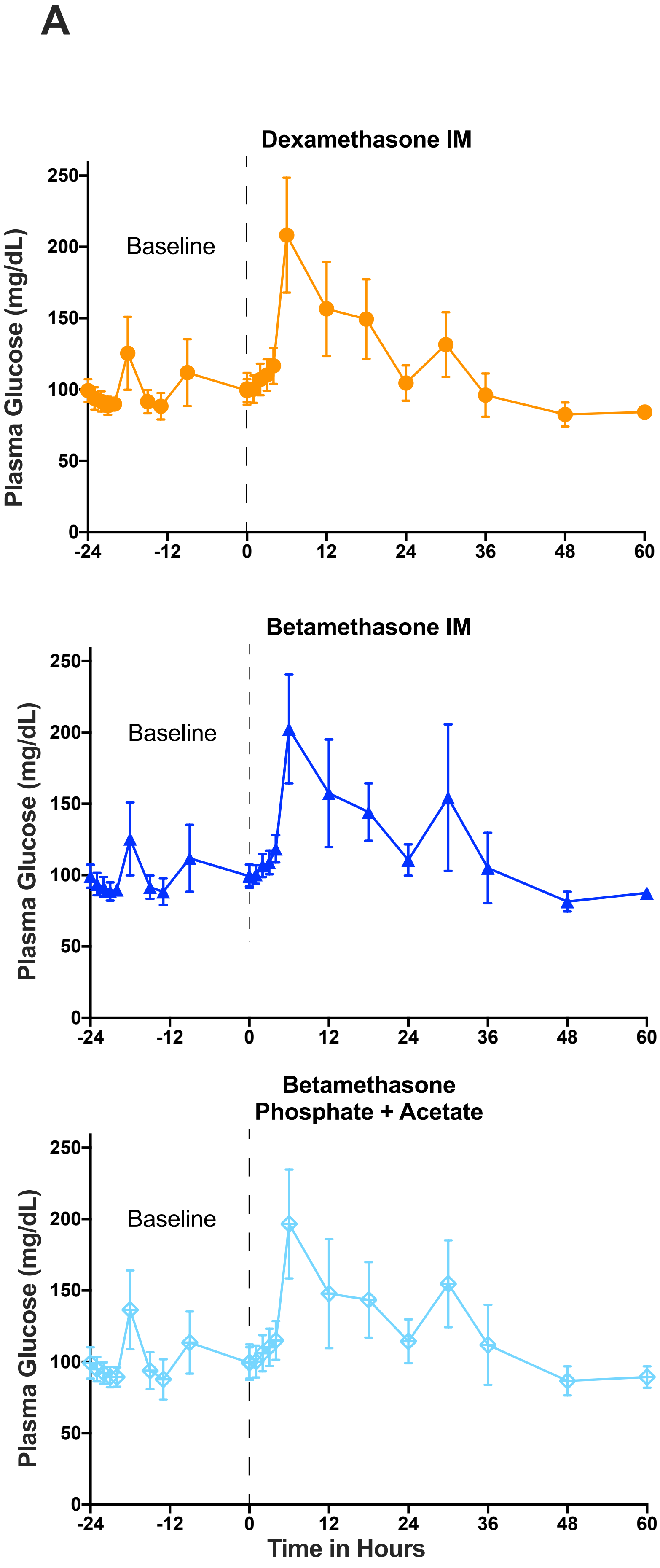
A
Dexamethasone IM
Dexamethasone Oral
Betamethasone IM
Betamethasone Oral
Betamethasone
Phosphate + Acetate
Baseline
Plasma Glucose (mg/dL)
Time in Hours
0
50
100
150
200
250
-24
-12
0
12
24
36
48
60

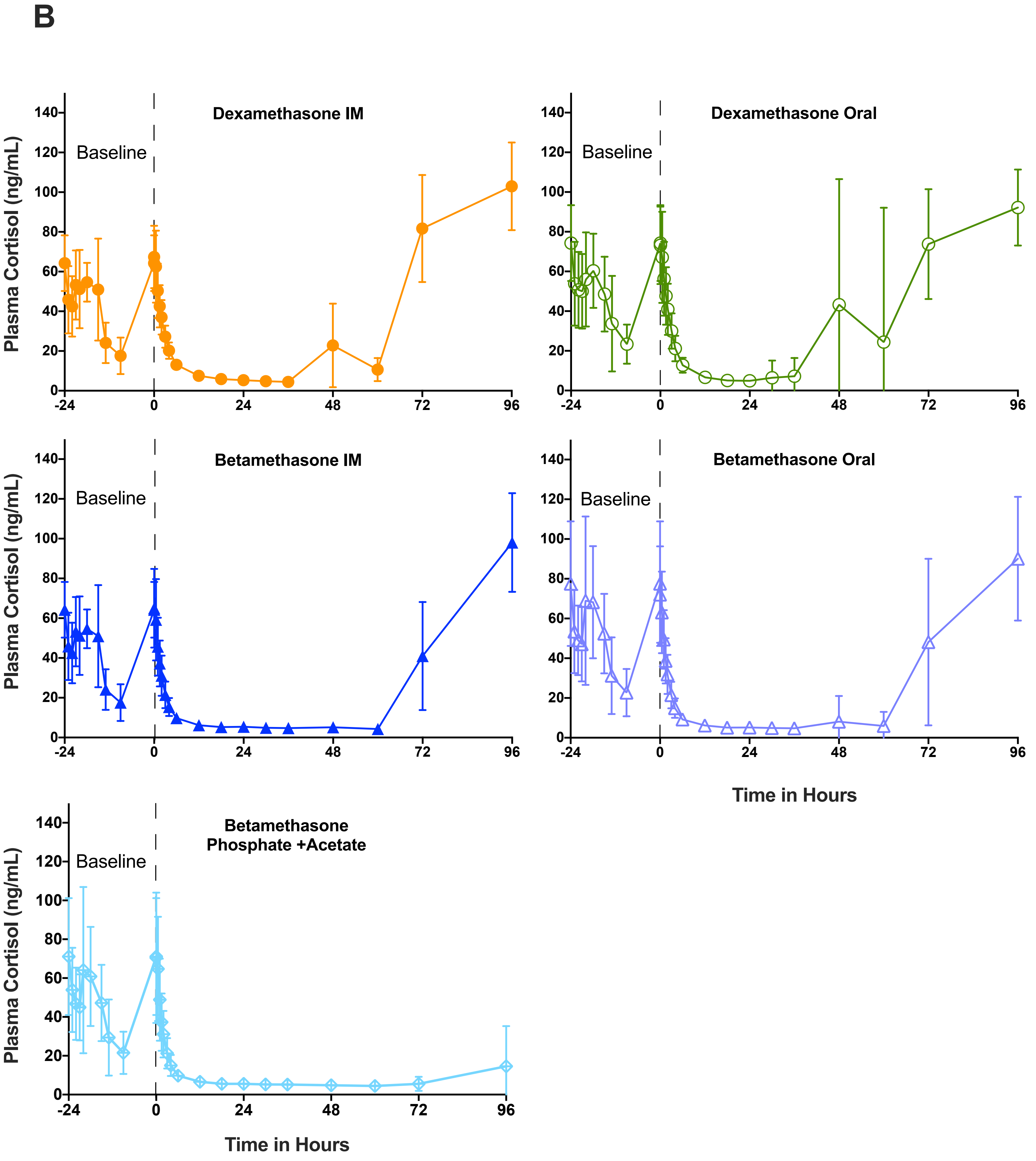
B
Dexamethasone IM
Dexamethasone Oral
Betamethasone IM
Betamethasone Oral
Betamethasone Phosphate +Acetate
Baseline
Plasma Cortisol (ng/mL)
Time in Hours
0
20
40
60
80
100
120
140
-24
0
24
48
72
96

Fig S1: Baseline and treatment response curves for (a) plasma glucose and (b) plasma cortisol. Group means ± 1 SD are given for each of the IM or Oral treatments that delivered 6 mg of dexamethasone or betamethasone.
