# Supplementary material for: Pharmacokinetics and Pharmacodynamics of Intramuscular and Oral Betamethasone and Dexamethasone in Reproductive Age Women in India
Source: Clin Transl Sci. 2019 Dec 13;13(2):391–9. doi: 10.1111/cts.12724 (PMC7070803; doi:10.1111/cts.12724)
Supplement: Supplementary file 2 — Figure S2. Baseline and treatment response curves for (a) blood neutrophils, (b) basophils, (c) CD3CD4 lymphocytes, and (d) CD3CD8 lymphocytes. Group means ± 1 SD are given for each of the IM or Oral treatments that delivered 6 mg of dexamethasone or betamethasone. [file CTS-13-391-s002.pdf]

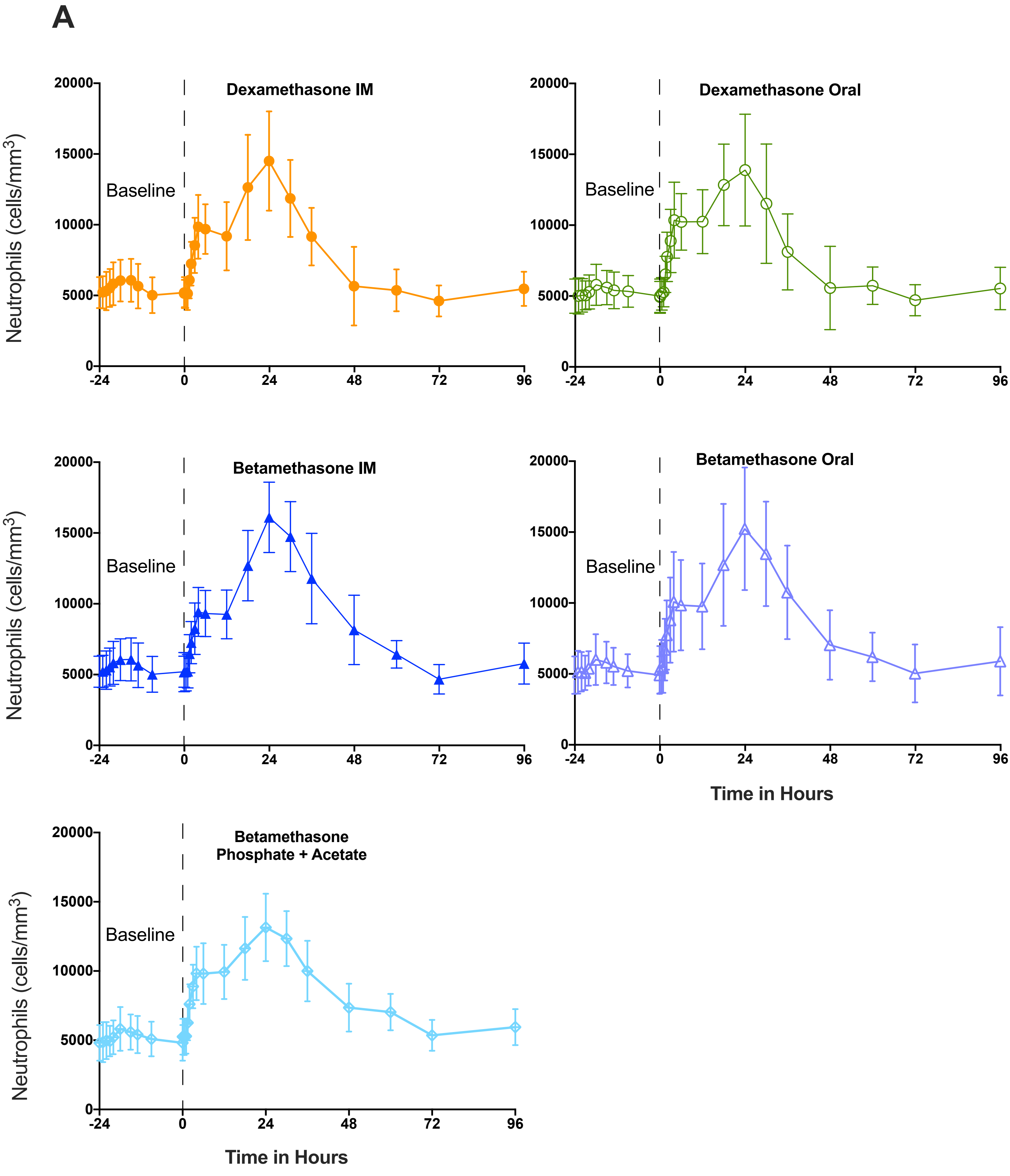
A
Dexamethasone IM
Dexamethasone Oral
Betamethasone IM
Betamethasone Oral
Betamethasone
Phosphate + Acetate
Baseline
Neutrophils (cells/mm3)
Time in Hours
20000
15000
10000
5000
0
-24
0
24
48
72
96

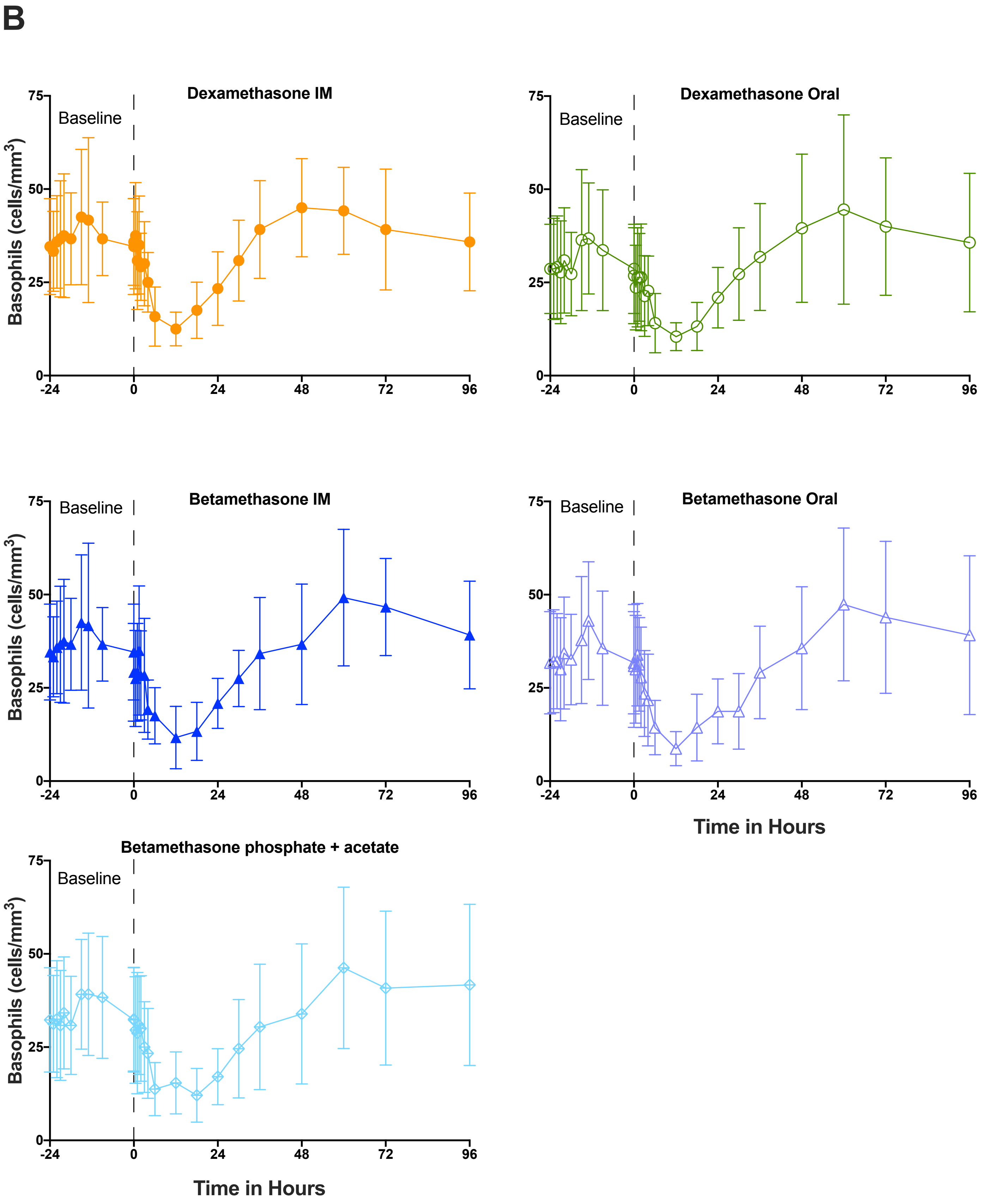

B
Dexamethasone IM
Dexamethasone Oral
Betamethasone IM
Betamethasone Oral
Betamethasone phosphate + acetate
Baseline
Basophils (cells/mm³)
Time in Hours
75
50
25
0
-24
0
24
48
72
96

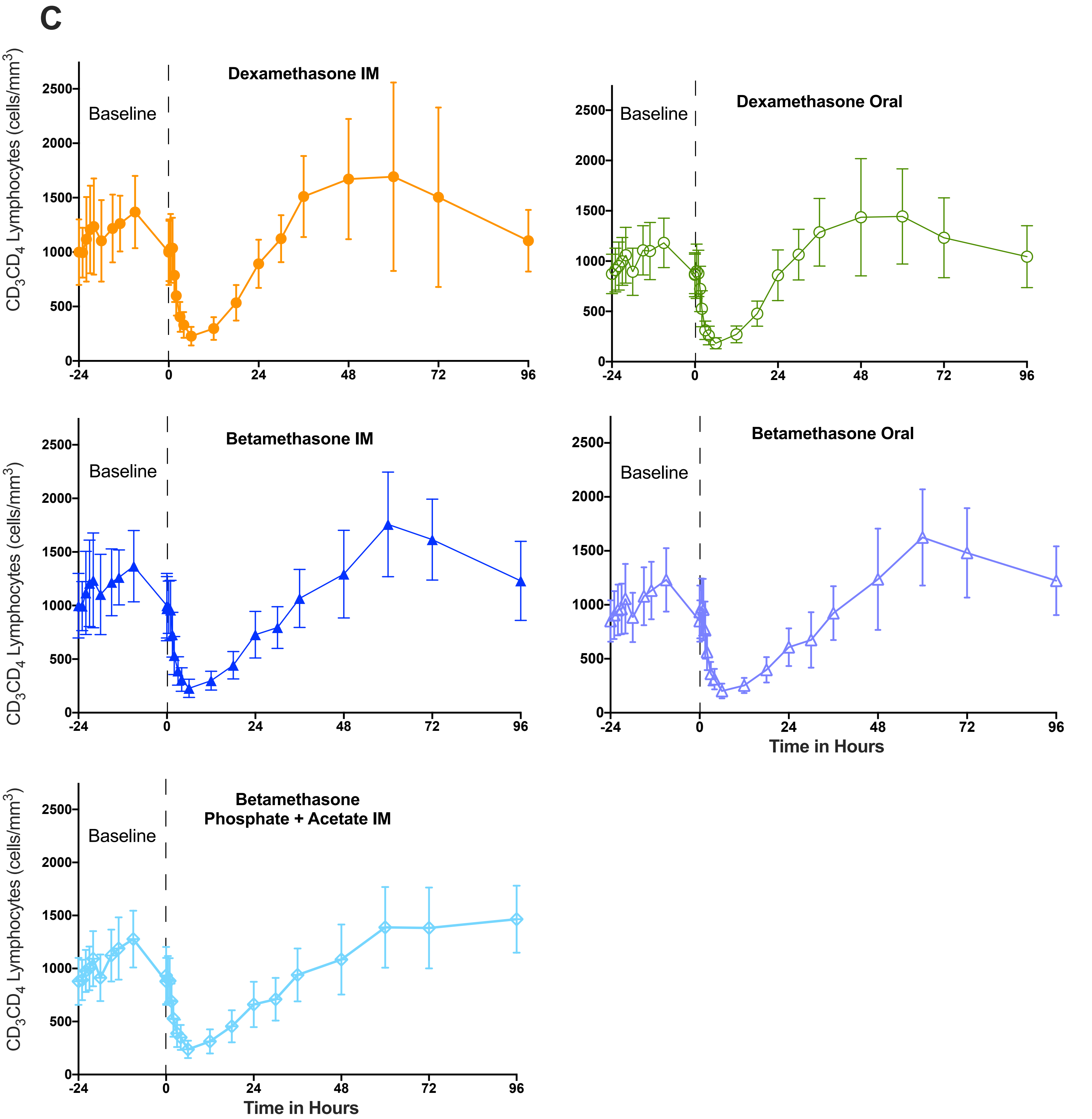
C
Dexamethasone IM
Baseline
Dexamethasone Oral
Baseline
Betamethasone IM
Baseline
Betamethasone Oral
Baseline
Betamethasone
Phosphate + Acetate IM
Baseline
CD_3CD_4 Lymphocytes (cells/mm^3)
Time in Hours
0
500
1000
1500
2000
2500
-24
0
24
48
72
96

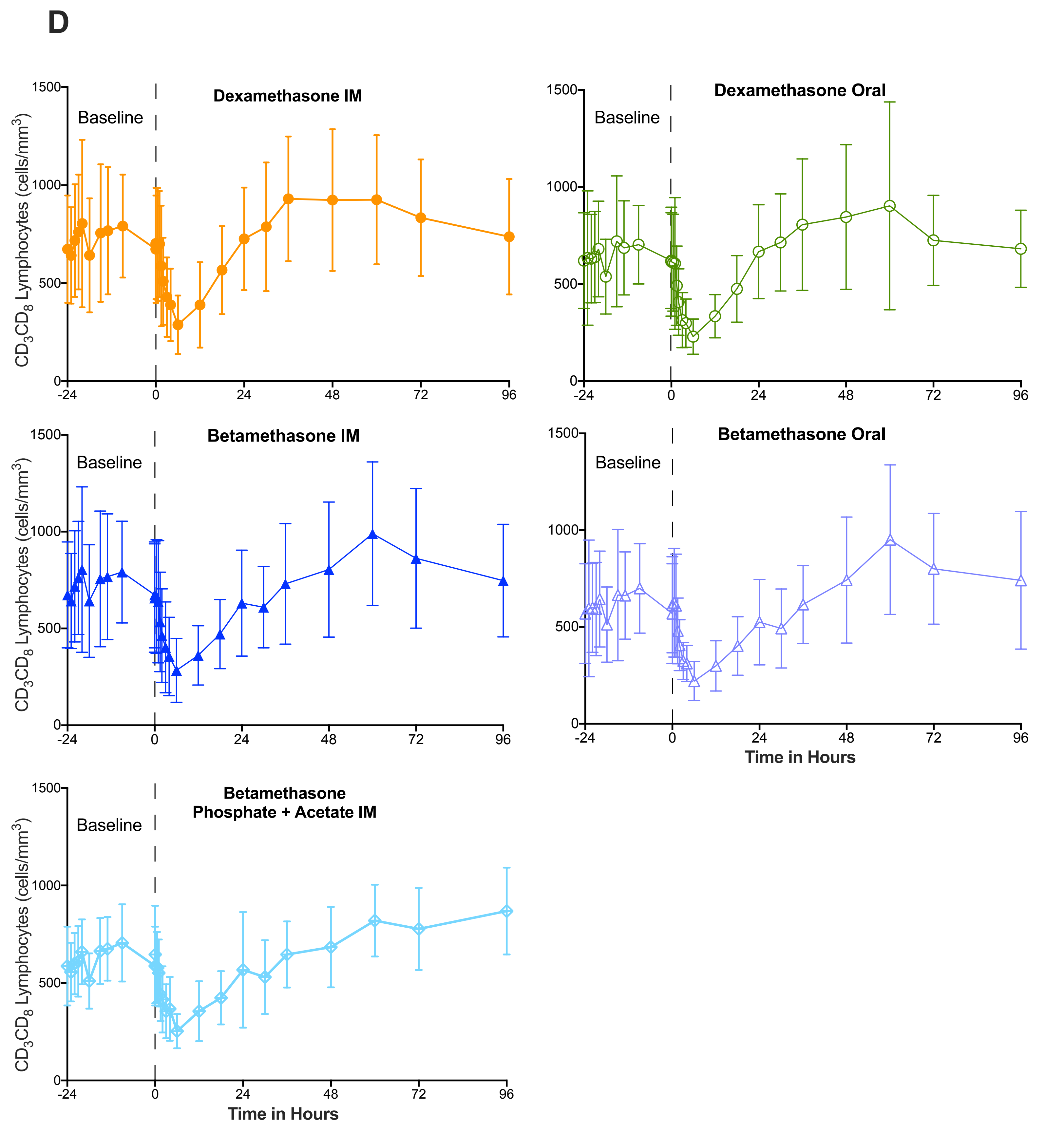
D
Dexamethasone IM
Dexamethasone Oral
Betamethasone IM
Betamethasone Oral
Betamethasone
Phosphate + Acetate IM
Baseline
CD_3CD_8 Lymphocytes (cells/mm^3)
Time in Hours
1500
1000
500
0
-24
0
24
48
72
96

Fig S2: Baseline and treatment response curves for (a) blood neutrophils, (b) basophils, (c) CD_3CD_4 lymphocytes, and (d) CD_3CD_8 lymphocytes. Group means ± 1 SD are given for each of the IM or Oral treatments that delivered 6 mg of dexamethasone or betamethasone.
