# Supplementary material for: Pharmacokinetics and Pharmacodynamics of Intramuscular and Oral Betamethasone and Dexamethasone in Reproductive Age Women in India
Source: Clin Transl Sci. 2019 Dec 13;13(2):391–9. doi: 10.1111/cts.12724 (PMC7070803; doi:10.1111/cts.12724)
Supplement: Supplementary file 3 — Table S1. Corticosteroids and source used for the study. [file CTS-13-391-s003.pdf]

Table S1: Source of Corticosteroids used for study

Corticosteroid	Route of Treatment	Format	Source	% of label claim
Dexamethasone $NaPO_4$	IM	4mg/ml-8ml Amp.	Dexalab-Laborate Pharma India	103%
Betamethasone $NaPO_4$	IM	4mg/ml	Betnsol®-GalaxoSmithKlein-India	97%
Betamethasone $NaPO_4$ + Betamethasone Ac	IM	6mg/ml	Celestone® Soluspan-MERCK, USA	101%
Dexamethasone $NaPO_4$	PO	0.5mg tabs	Dexona®- Zydas Alidac, India	108%
Betamethasone $NaPO_4$	PO	0.5mg tabs	Betnsol®-GalaxoSmithKlein-India	102%
