# Supplementary material for: Pharmacokinetics and Pharmacodynamics of Intramuscular and Oral Betamethasone and Dexamethasone in Reproductive Age Women in India
Source: Clin Transl Sci. 2019 Dec 13;13(2):391–9. doi: 10.1111/cts.12724 (PMC7070803; doi:10.1111/cts.12724)
Supplement: Supplementary file 4 — Table S2. Demographics of study population. [file CTS-13-391-s004.pdf]

Table S2: Demographics of study population (Mean ± SD)

Treatment	IM Dexamethasone phosphate	IM Betamethasone phosphate	IM Betamethasone phosphate plus betamethasone acetate	Oral Dexamethasone phosphate	Oral Betamethasone phosphate
Asian, Female (N)*	12	12	24	22	23
Age (years)	32.2 ± 4.9	32.2 ± 4.9	31.6 ± 4.0	31.6 ± 3.2	31.4 ± 3.9
Height (cm)	152.3 ± 5.1	152.3 ± 5.1	152.8 ± 5.23	153.5 ± 4.3	153.3 ± 5.6
Weight (kg)	56.7 ± 4.2	56.7 ± 4.2	55.9 ± 4.4	56.3 ± 4.0	57.7 ± 4.5
BMI (kg/m^2)	24.4 ± 0.9	24.4 ± 0.9	23.9 ± 1.2	23.9 ± 1.3	24.5 ± 0.6

*Demographics are for women randomized to the 5 treatment groups, but excluding those without PK or PD profiles. Each woman is included in 2 groups.
