# Supplementary material for: Pharmacokinetics and Pharmacodynamics of Intramuscular and Oral Betamethasone and Dexamethasone in Reproductive Age Women in India
Source: Clin Transl Sci. 2019 Dec 13;13(2):391–9. doi: 10.1111/cts.12724 (PMC7070803; doi:10.1111/cts.12724)
Supplement: Supplementary file 5 — Table S3. Baseline cortisol values and Percent change from Period 1 to Period 2. [file CTS-13-391-s005.pdf]

Table S3: Mean ±1 SD Baseline Cortisol Values and Percent Change from Period 1 to Period 2 Baseline Values by Treatment in Period 1

Treatment in Period 1	IM Dexamethasone phosphate	IM Betamethasone phosphate	IM Betamethasone phosphate plus betamethasone acetate	Oral Dexamethasone phosphate	Oral Betamethasone phosphate
**Cortisol**					
Period 1 Hr 0 (μg/mL)	69.1 ± 13.8	68.1 ± 16.8	84.3 ± 40.1	73.7 ± 18.7	81.2 ± 28.7
Period 2 Hr 0 (μg/mL	62.0 ± 23.7	65.7 ± 18.7	62.7 ± 13.9	62.4 ± 16.7	61.5 ± 23.7
%-Change from Period 1 to Period 2	-11.8 ± 24.2	0.2 ± 33.1	-14.3 ± 33.5	-13.9 ± 18.3	-23.5 ± 19.7
P-value	0.637				

Note: P-value is testing for differences among the five treatments from the analysis of covariance model with terms for the Period 1, Hr 0 value and treatment.
