# Supplementary material for: Pharmacokinetics and Pharmacodynamics of Intramuscular and Oral Betamethasone and Dexamethasone in Reproductive Age Women in India
Source: Clin Transl Sci. 2019 Dec 13;13(2):391–9. doi: 10.1111/cts.12724 (PMC7070803; doi:10.1111/cts.12724)
Supplement: Supplementary file 6 — Table S4. Pharmacodynamic Values for Neutrophils, Basophils, CD3CD4 and CD3CD8 lymphocytes in blood following 6mg corticosteroids treatments. [file CTS-13-391-s006.pdf]

Table S4: Summary Statistics (Mean ± SD or Median, 25 - 75percentiles) of Pharmacodynamic Parameters for Neutrophils, Basophils, CD3CD4 and CD3CD8 Lymphocytes in Blood Following a Single Dose of 6mg Corticosteroid.

Treatment	IM Dexamethasone phosphate	IM Betamethasone phosphate	IM Betamethasone phosphate plus betamethasone acetate	Oral Dexamethasone phosphate	Oral Betamethasone phosphate
Number of PD curves	12	12	24	22	23
**Neutrophils**					
Hr 0 (/mm^3)	5137 ± 971	5174 ± 1382	5254 ± 1290	4933 ± 1097	5296 ± 1674
Max (/mm^3)	14513 ± 3494	16123 ± 2485	13428 ± 2263	14406 ± 3622	15305 ± 4302
t_{max} (hours)	24.0<br>24.0 - 24.0	24.0<br>24.0 - 24.0	24.0<br>24.0 - 24.0	24.0<br>18.0 - 24.0	24.0<br>24.0 - 24.0
Number Censored	1	0	8	0	3
Rebound Time (hours)	49.4<br>45.5 - 55.4	63.5<br>55.1 - 68.9	74.6<br>55.0 - >96	46.1<br>42.7 - 55.9	70.6<br>49.9 - 78.1
Increase $AUEC_{RT}$ ($x10^3 \cdot hr/mm^3$)	228<br>161 - 264	299<br>259 - 368	267<br>228 - 331	203<br>169 - 268	303<br>226 - 377
**Basophils**					
Hr 0 (/mm^3)	35.8 ± 11.7	29.2 ± 13.1	32.5 ± 13.9	26.8 ± 12.9	30.9 ± 16.5
Min (/mm^3)	10.8 ± 2.9	10.0 ± 7.4	9.6 ± 5.5	8.6 ± 4.7	7.4 ± 4.5
t_{min} (hours)	6.0<br>2.5 - 9.5	12.0<br>6.0 - 12.0	6.0<br>5.0 - 12.0	4.5<br>1.5 - 12.0	6.0<br>4.0 - 12.0
Number Censored	0	0	1	0	0
Rebound Time (hours)	26.3<br>24.0 - 35.7	30.0<br>24.0 - 38.6	27.8<br>25.5 - 43.3	24.0<br>22.7 - 28.0	30.0<br>24.0 - 42.4
Decrease $AUEC_{RT}$ (hr/mm^3)	473<br>251 - 708	553<br>326 - 925	471<br>308 - 798	259<br>170 - 638	493<br>320 - 893
**CD3CD4 Lymphocytes**					
Hr 0 (/mm^3)	1011 ± 277	971 ± 299	935 ± 269	864 ± 218	934 ± 245
Min (/mm^3)	227 ± 86	220 ± 86	235 ± 81	175 ± 61	196 ± 65
t_{min} (hours)	6.0<br>6.0 - 6.0	6.0<br>6.0 - 6.0	6.0<br>6.0 - 6.0	6.0<br>6.0 - 6.0	6.0<br>6.0 - 6.0
Number Censored	0	0	0	1	0
Rebound Time (hours)	25.6<br>23.6 - 31.7	40.9<br>39.4 - 43.9	42.2<br>31.6 - 44.8	25.4<br>23.2 - 30.8	42.2<br>29.8 - 44.9

Table S4 - continued

Treatment	IM Dexamethasone phosphate	IM Betamethasone phosphate	IM Betamethasone phosphate plus betamethasone acetate	Oral Dexamethasone phosphate	Oral Betamethasone phosphate
Number of PD curves	12	12	24	22	23
Decrease $AUEC_{RT}$ ($x10^3 \cdot hr/mm^3$)	17.4<br>12.1 - 23.4	21.8<br>15.7 - 28.4	19.4<br>16.7 - 23.4	15.5<br>10.9 - 18.8	20.3<br>16.4 - 22.5
**CD3CD8 Lymphocytes**					
Hr 0 ($/mm^3$)	702 ± 284	657 ± 280	646 ± 250	616 ± 281	615 ± 248
Min ($/mm^3$)	287 ± 150	276 ± 171	250 ± 88	213 ± 97	213 ± 81
t_{min} (hours)	6.0<br>6.0 - 6.0	6.0<br>6.0 - 6.0	6.0<br>6.0 - 6.0	6.0<br>6.0 - 6.0	6.0<br>6.0 - 6.0
Number Censored	0	0	0	0	1
Rebound Time (hours)	22.6<br>20.5 - 24.0	24.0<br>23.4 - 32.0	28.6<br>22.0 - 34.6	23.1<br>21.3 - 24.8	29.8<br>22.1 - 39.9
Decrease $AUEC_{RT}$ ($x10^3 \cdot hr/mm^3$)	5.7<br>4.6 - 7.4	7.7<br>5.6 - 9.1	6.5<br>5.5 - 7.2	5.9<br>4.4 - 8.0	6.4<br>4.6 - 8.2
