# Supplementary material for: Pharmacokinetics and Pharmacodynamics of Intramuscular and Oral Betamethasone and Dexamethasone in Reproductive Age Women in India
Source: Clin Transl Sci. 2019 Dec 13;13(2):391–9. doi: 10.1111/cts.12724 (PMC7070803; doi:10.1111/cts.12724)
Supplement: Supplementary file 7 — Table S5. Neutrophil counts and Percent change from Period 1 to Period 2. [file CTS-13-391-s007.pdf]

Table S5: Mean ±1 SD Baseline Neutrophil Counts and Percent Change from Period 1 to Period 2.

| Treatment in Period 1 | IM Dexamethasone phosphate | IM Betamethasone phosphate | IM Betamethasone phosphate plus betamethasone acetate | Oral Dexamethasone phosphate | Oral Betamethasone phosphate |
|---|---|---|---|---|---|
| **Neutrophils** | | | | | |
| Period 1 Hr 0 (/mm$^3$) | 5617 ± 901 | 4882 ± 1463 | 4762 ± 1121 | 4715 ± 1008 | 5152 ± 1411 |
| Period 2 Hr 0 (/mm$^3$) | 5467 ± 1363 | 4657 ± 843 | 5192 ± 1234 | 5926 ± 1865 | 5043 ± 1337 |
| %-Change from Period 1 to Period 2 | -3.0 ± 17.9 | -0.1 ± 26.4 | 13.1 ± 32.6 | 26.3 ± 31.9 | 1.0 ± 22.8 |
| P-value | 0.265 | | | | |

Note: P-value is testing for differences among the five treatments from the analysis of covariance model with terms for the Period 1, Hr 0 value and treatment.
