# Supplementary material for: Pharmacokinetics and Pharmacodynamics of Intramuscular and Oral Betamethasone and Dexamethasone in Reproductive Age Women in India
Source: Clin Transl Sci. 2019 Dec 13;13(2):391–9. doi: 10.1111/cts.12724 (PMC7070803; doi:10.1111/cts.12724)
Supplement: Supplementary file 8 — Supplemental Figure Legends. Supplemental Figure Legends. [file CTS-13-391-s008.pdf]

Supplemental Figure and Tables:

Table S1: Corticosteroids and source used for the study

Table S2: Demographics of study population

Fig S1: Baseline and treatment response curves for (a) plasma glucose and (b) plasma cortisol. Group means $\pm$ 1 SD are given for each of the IM or Oral treatments that delivered 6 mg of dexamethasone or betamethasone.

Table S3: Baseline cortisol values and Percent change from Period 1 to Period 2.

Fig S2: Baseline and treatment response curves for (a) blood neutrophils, (b) basophils, (c) CD_3CD_4 lymphocytes, and (d) CD_3CD_8 lymphocytes. Group means $\pm$ 1 SD are given for each of the IM or Oral treatments that delivered 6 mg of dexamethasone or betamethasone.

Table S4: Pharmacodynamic Values for Neutrophils, Basophils, CD_3CD_4 and CD_3CD_8 lymphocytes in blood following 6mg corticosteroids treatments.

Table S5: Neutrophil counts and Percent change from Period 1 to Period 2.
