# Supplementary material for: Pharmacokinetics and Pharmacodynamics of Intramuscular and Oral Betamethasone and Dexamethasone in Reproductive Age Women in India
Source: Clin Transl Sci. 2019 Dec 13;13(2):391–9. doi: 10.1111/cts.12724 (PMC7070803; doi:10.1111/cts.12724)
Supplement: Supplementary file 9 — Supplemental cover Page. Supplemental cover page. [file CTS-13-391-s009.pdf]

Supplement to:

Pharmacokinetics and Pharmacodynamics of intramuscular and oral betamethasone and dexamethasone in reproductive age women in India

Alan H. Jobe
Division of Pulmonary Biology
Cincinnati Children's Hospital Medical Center
University of Cincinnati, Cincinnati, OH

Mark A. Milad
Milad Pharmaceutical Consulting LLC,
Plymouth, Michigan, USA

Thomas Peppard
Certara, Inc,
Princeton, NJ, USA

William J. Jusko
State University of New York
School of Pharmacy and Pharmaceutical Sciences
University of Buffalo
Buffalo, NY, USA

Corresponding Author:
Alan H. Jobe, MD, PhD
Cincinnati Children's Hospital
3333 Burnet Avenue
Cincinnati, OH 45229
513-636-8563
Alan.Jobe@cchmc.org
